# Nucleocytoplasmic Shuttling of the TACC Protein Mia1p/Alp7p Is Required for Remodeling of Microtubule Arrays during the Cell Cycle

**DOI:** 10.1371/journal.pone.0006255

**Published:** 2009-07-16

**Authors:** Yuen Chyao Ling, Aleksandar Vjestica, Snezhana Oliferenko

**Affiliations:** Temasek Life Sciences Laboratory, Singapore, Singapore; University of Edinburgh, United Kingdom

## Abstract

Microtubule arrays are remodeled as cells proceed through the cell cycle. It is important to understand how remodeling is regulated in time and space. In fission yeast, the conserved microtubule associated TACC/TOG complex plays an important role in organizing microtubules throughout the cell cycle. Here we show that this complex undergoes nucleocytoplasmic shuttling through the nuclear import and export signals located in the TACC protein Mia1p/Alp7p. When the Crm1p-dependent nuclear export signal of Mia1p is disabled, Mia1p accumulates in the nucleus while its partner protein Alp14p/TOG is restricted to the cytoplasm. This leads to defects in assembly of both interphase arrays and the mitotic spindle. Artificial targeting of Alp14p to the nucleus partially rescues the mitotic spindle defects caused by lack of Mia1p nuclear export. Interestingly, the nuclear export sequence of Mia1p appears to overlap with the Alp14p binding site. We propose that intricate regulation of the subcellular distribution of TACC/TOG complexes drives microtubule array remodeling as cells progress through the cell cycle.

## Introduction

Microtubules are dynamic polymers that often function as higher order arrays of different geometries that form in response to cell cycle and environmental cues. In interphase, cytoplasmic microtubule arrays sustain specific cell morphology and function. As cells enter mitosis, microtubules are reorganized to form a mitotic spindle. In “open” mitosis of higher eukaryotes the nuclear envelope (NE) breaks down enabling microtubules to capture kinetochores. In many organisms, such as fission yeast *Schizosaccharomyces pombe* (*S. pombe*), the NE stays intact so spindles are assembled from tubulin and microtubule-associated proteins (MAPs) that are imported from the cytoplasm through the nuclear pore complexes. Such mitosis is called “closed”. It appears that karyopherins involved in nuclear transport together with the small GTPase Ran play an important role in spindle assembly in both systems. In animal cells, a Ran-GTP mediated release of the microtubule regulators, such as TPX2 [1], NUMA [2], [3] and HURP [4], [5] from complexes with karyopherins allows spindle assembly. In *S. pombe*, Ran-GTP promotes nuclear accumulation of an evolutionary conserved MAP complex that consists of the transforming acidic coiled coil (TACC) protein Mia1p/Alp7p (which appears to be a direct Ran target) and the TOG protein Alp14p [6]. It is unclear whether nucleocytoplasmic shuttling occurs throughout the cell cycle and why this complex accumulates in the nucleus only at mitotic onset. Here we show that Mia1p/Alp7p shuttles in and out of the nucleus during interphase by utilizing a nuclear import sequence (NLS) and a nuclear export sequence (NES). When the NES is mutated Mia1p accumulates in the nucleus. This leads to profound microtubule abnormalities at all stages of cell cycle, including mitosis. We link the spindle-related phenotypes to deficient nuclear accumulation of the Mia1p/Alp7p partner, Alp14p. Our results underscore the importance of spatiotemporal regulation of the activity and availability of MAPs for proper microtubule array formation.

## Results

### Mia1p is exported from the nucleus via a Crm1p-dependent NES

**Figure 1:**
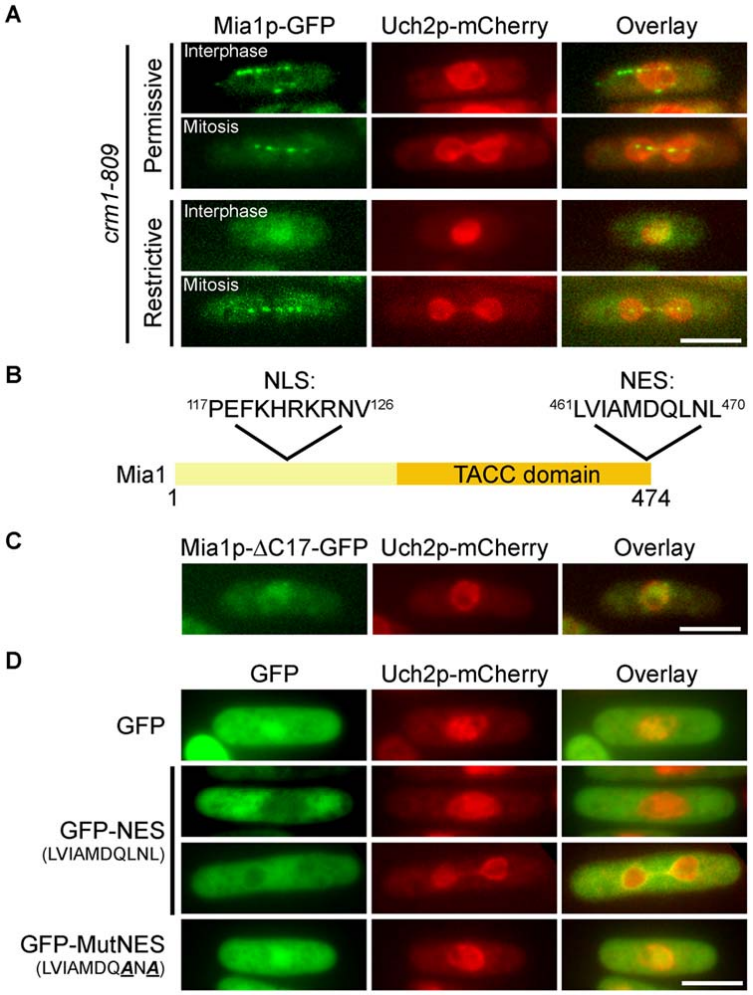
Mia1p is exported from the nucleus via a Crm1p-dependent NES. (A) Mia1p-GFP localized to mitotic spindles and interphase microtubules in *crm1-809* Uch2p-mCherry expressing cells at 36°C but only to the nucleus at 18°C. (B) Positions of predicted NLS and NES on Mia1p. (C) Mia1p-ΔC17-GFP is enriched in the nucleus in interphase cells. (D) GFP-NES but not MutNES is excluded from the nucleus. Shown are single maximum intensity reconstructions of live cells. Scale bars = 5 µm.

At mitotic entry, Mia1p accumulates in the nucleus, while during interphase it is restricted to the cytoplasm. We wondered what is the mechanism that drives its differential compartmentalization during the cell cycle. The ELM algorithm [7] predicts a putative leucine-rich NES motif at the extreme C-terminus of Mia1p that is recognized by a major cellular exportin Crm1p. Thus, we checked the localization of Mia1p-GFP in cells containing a mutant allele of *crm1*, *crm1-809* [8] and an NE marker, Uch2p-mCherry. At the permissive temperature of 36°C, Mia1p-GFP in interphase cells localized along microtubules in the cytoplasm. However, Mia1p-GFP accumulated in the nuclei of interphase *crm1-809* cells at the restrictive temperature of 18°C (nuclear Mia1p-GFP signal increases ∼90%, n = 50 cells, p<0.01 upon temperature downshift). Its mitotic localization to the spindle pole bodies (SPBs), kinetochores and along the spindle remained unchanged (Fig. 1A). An obligate partner of Mia1p, Alp14p/TOG colocalized with Mia1p in the nuclei of *crm1-809* cells at 18°C (Fig. S1A), suggesting that the entire TACC/TOG complex shuttles in and out of the nucleus. We confirmed these observations by inhibiting Crm1p function through treatment with leptomycin B (data not shown).

**Figure 2:**
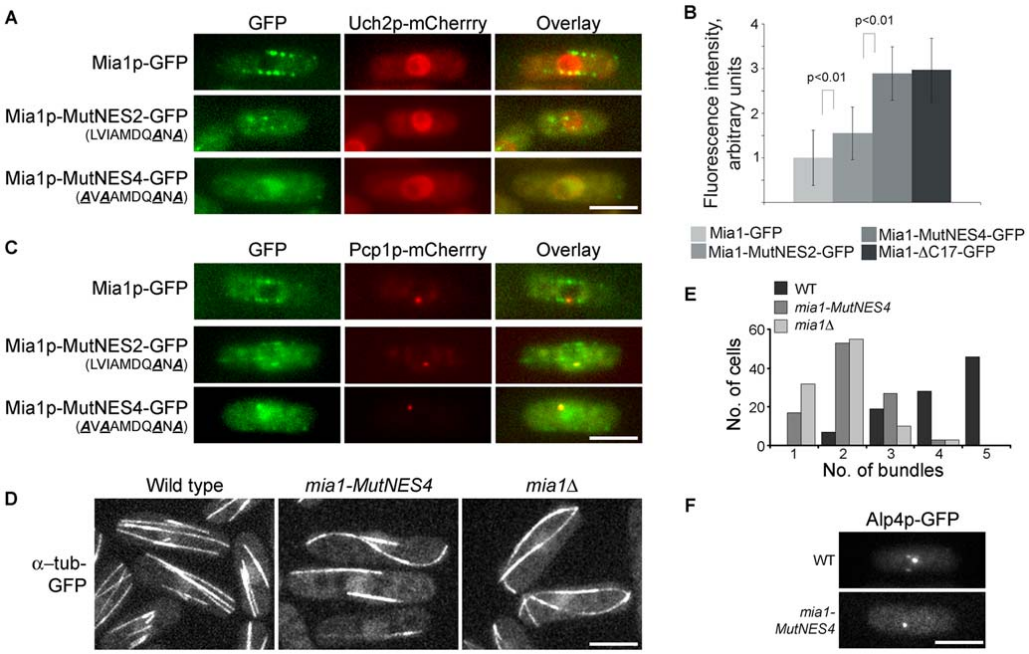
Nuclear retention of Mia1p leads to abnormal organization of interphase microtubule arrays. (A) Localization of Mia1p-GFP in interphase cells, with Uch2-mCherry as an NE marker. (B) Relative intensities of nuclear Mia1p-GFP fluorescence in interphase cells of various *mia1* mutant backgrounds (n = 100 cells). (C) Localization of Mia1p-GFP in interphase cells, with Pcp1p-mCherry as an SPB marker. (D) *mia1-MutNES4* cells expressing α-tubulin-GFP exhibited fewer interphase microtubule bundles, similar to *mia1*Δ cells. (E) Quantification of the number of interphase microtubule bundles in wild type, *mia1-MutNES4* and *mia1*Δcells (n = 100 cells). (F) Alp4p-GFP is detected as several distinct dots in wild type but not in *mia1-MutNES4* cells treated with MBC. Shown are single maximum intensity reconstructions of live cells. Scale bars = 5 µm.

Deletion of the last 17 amino acids (Mia1p-CΔ17) comprising the putative NES led to nuclear accumulation of Mia1p during interphase (Fig. 1B and C, quantified in Fig. 2B). When fused to GFP, this sequence (LVIAMDQLNL) was sufficient to deplete GFP from the nucleus throughout the cell cycle (Fig. 1D and Fig. S1B). Replacement of last two leucine residues with alanine (LVIAMDQANA) restored the ubiquitous localization of GFP to both nucleus and cytoplasm (Fig. 1D). Thus, we concluded that Mia1p contains a Crm1p-dependent NES and shuttles between the nucleus and cytoplasm, even during interphase.

### Nuclear retention of Mia1p during interphase leads to pronounced defects in assembly of cytoplasmic microtubule arrays

To specifically disrupt NES function we introduced LVIAMDQANA and AVAAMDQANA (denoted Mia1p-MutNES2 and Mia1p-MutNES4, respectively) mutations to the *mia1* open reading frame at its chromosomal locus. Uch2p-mCherry and Pcp1p-mCherry were used as NE and SPB markers. We observed a relative equilibration of Mia1p-MutNES2-GFP fluorescence between the nucleus and cytoplasm of interphase cells and reduced localization of the mutant protein to the linear microtubule arrays. On the other hand, Mia1p-MutNES4-GFP constitutively accumulated in the nucleus and was completely excluded from interphase microtubule arrays (Fig. 2A, B and C), similar to Mia1p-CΔ17-GFP. We concluded that Leu/Ile residues 461, 463, 468 and 470 contributed to NES function *in vivo* and therefore chose the Mia1p-MutNES4 mutant for further characterization.

We then evaluated the functional consequences of Mia1p retention in the nucleus during interphase. First, we expressed α-tubulin-GFP in cells containing untagged Mia1p-MutNES4 mutant protein as the sole source of Mia1p. We observed striking abnormalities in the organization of interphase microtubule arrays (Fig. 2D). Interphase Mia1p-MutNES4 cells contained fewer microtubule bundles and microtubules often curved around cell tips, similar to *mia1*Δ cells (Fig. 2E, Movie S1, compare to the wild type, Movie S2). Upon addition of the microtubule-depolymerizing drug MBC, interphase microtubule bundles depolymerize to short stubs, usually attached to the NE. These sites are also enriched in the γ-tubulin complex proteins, such as Alp4p and Mto1p [9]. Similar to the *mia1*Δ phenotype, Alp4p-GFP did not exhibit multiple dots around the NE and instead localized only to the SPB in Mia1p-MutNES4 cells (Fig. 2F), indicating that microtubule bundles are not properly organized at the NE. These results suggest a lack of Mia1p function in the cytoplasm and emphasize the necessity for the active nuclear export of Mia1p at the end of mitosis to ensure remodeling of microtubule arrays as cells proceed into the new cell cycle.

### Mia1p NES mutation confers abnormalities on mitotic spindle organization

**Figure 3:**
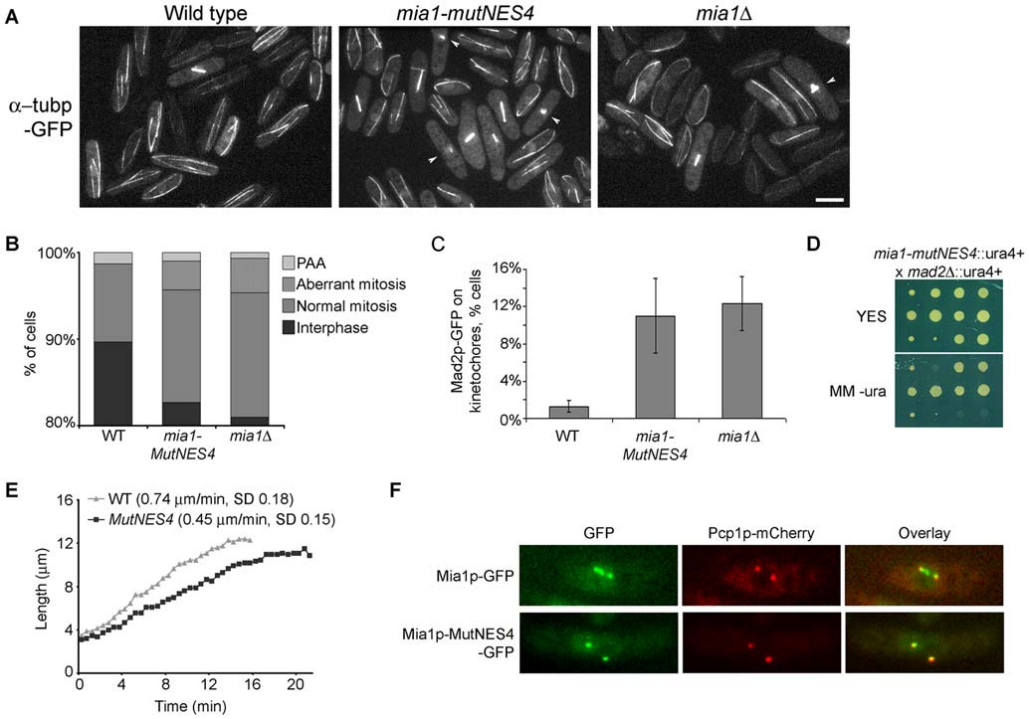
Mutation in the Mia1p NES triggers mitotic spindle abnormalities. (A) *mia1-MutNES4* cells exhibit frequent spindle abnormalities such as monopolar and broken spindles. Shown are representative fields of wild type, *mia1-MutNES4* and *mia1*Δcells expressing α-tubulin-GFP. Abnormal spindles are indicated by white arrowheads. (B) Percentages of aberrant mitoses in asynchronous cell populations (n = 300 cells). PAA, Post-anaphase microtubule arrays. (C) Percentages of cells exhibiting Mad2p-GFP at kinetochores (n = 300 cells). (D) *mia1-MutNES4 mad2*Δ cells are severely compromised for growth. Shown are three sets of segregants (from top to bottom: tetratype, parental ditype and non-parental ditype) from tetrads obtained from a cross between *mia1-MutNES4* and *mad2*Δ cells, grown on YES agar (upper panel) and later replicated onto minimal medium lacking uracil (lower panel). Both the *mia1+* and *mad2+* genes were mutated using *ura4+* gene. Note the smaller colony size in double mutants. (E) *mia1-MutNES4* cells exhibit a decreased anaphase elongation rate. (F) Unlike Mia1p-GFP, Mia1p-MutNES4-GFP does not localize to kinetochores during mitosis. Pcp1p-mCherry was used as an SPB marker. Scale bars = 5 µm.

To our surprise, we noticed an increased proportion of mitotic cells in the Mia1p-MutNES4 strain, as well as many instances of mitotic spindle abnormalities including monopolar and broken spindles (Fig. 3A and B). Again, this phenotype was similar to that of *mia1*Δ cells [10]. An abnormally large fraction of Mia1p-MutNES4 cells (∼10%) showed the spindle assembly checkpoint (SAC) protein Mad2p present at kinetochores, indicative of faulty microtubule/kinetochore interaction and metaphase delay (Fig. 3C). To compare, Mad2p-GFP was found on kinetochores of ∼12% *mia1*Δ cells (Fig. 3C). Disabling of the SAC through deletion of *mad2* was lethal in an *mia1*Δ genetic background whereas cells carrying both *mad2*Δ and *mia1-mutNES4* mutations were severely compromised for growth (Fig. 3D). Thus, it appears that the SAC functions to correct mitotic abnormalities in an *mia1-mutNES4* background and suggests that a complete absence of Mia1p has more severe functional consequences than an NES mutation. Furthermore, we observed spindle breakage events (Movies S3 and S4) and a decreased anaphase spindle elongation rate as compared to wild type (Fig. 3E).

In mitotic cells, Mia1p normally localizes to the SPBs, kinetochores and along the spindle [10]. Interestingly, while Mia1p-MutNES4-GFP obviously localized to the nucleus during mitosis, it was largely depleted from the kinetochores and instead was restricted to the SPBs (Fig. 3F). Thus, it appears that mutations in the NES compromise localization of Mia1p to kinetochores in mitosis.

### Mutations to the NES compromise Mia1p interaction with Alp14p/TOG

**Figure 4:**
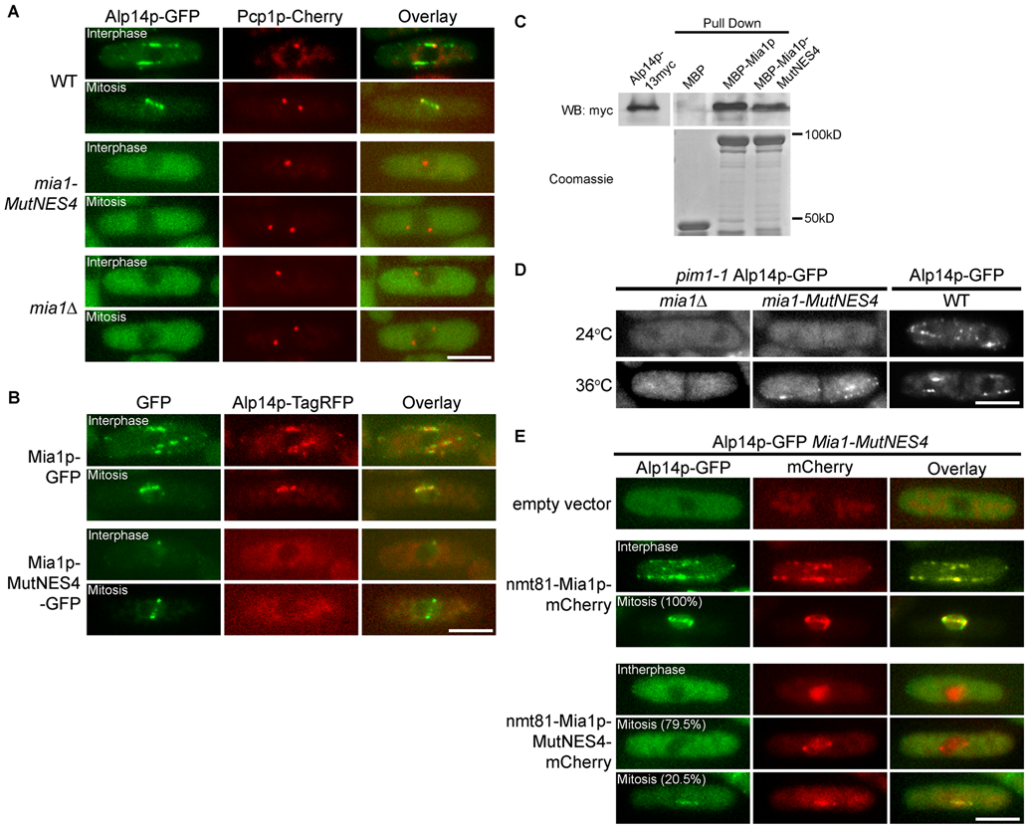
Interaction between Mia1p and Alp14p is affected by mutating the Mia1p NES. (A) Alp14p-GFP does not localize to microtubules in *mia1-MutNES4* and *mia1*Δ cells. Shown are single maximum intensity reconstructions of live cells expressing Alp14p-GFP and Pcp1-mCherry. (B) Mia1p-GFP and Alp14p-TagRFP are spatially separated in *mia1-MutNES4* cells. (C) MBP-Mia1p-MutNES4 showed weaker interaction with Alp14p-myc in pull-down assays, as compared to MBP-Mia1p. Alp14p-myc yeast lysates were extracted from *mia1*Δ cells and detected with anti-myc antibody. MBP-tagged proteins were detected by Coomassie staining. (D) When released from the nucleus using *pim1-1* mutation at 36°C, Mia1p-MutNES4 is capable of loading Alp14p-GFP on cytoplasmic microtubules. Localization of Alp14p-GFP in wild type septated cells at 24°C and 36°C is included as a control. Shown are single maximum intensity reconstructions of live cells. (E) Overexpression of Mia1p-MutNES4-mCherry partially restores nuclear accumulation and spindle localization of Alp14p-GFP during mitosis. Shown are single maximum intensity reconstructions of live cells. Scale bars = 5 µm.

Mitotic abnormalities in *mia1*Δ cells are caused largely by the inability of Alp14p to load onto the spindle [6]. In turn, Alp14p loads Mia1p on kinetochores [10]. We therefore wondered whether Alp14p localization was disrupted in *mia1-mutNES4* cells. We found that Alp14p-GFP was indeed depleted from microtubules in *mia1-mutNES4* cells, not only in the interphase but also during mitosis (Fig. 4A). Instead, Alp14p-GFP exhibited a diffuse cytosolic localization at all stages of the cell cycle, similar to *mia1*Δ cells (Fig. 4A). We verified that Alp14p co-localized with Mia1p but not with Mia1p carrying a mutated NES, by co-expressing Alp14p-TagRFP with either Mia1p-GFP or Mia1p-MutNES4-GFP fusion proteins (Fig. 4B).

Since the C-terminal half of Mia1p has been shown to interact with Alp14p [10], we wondered whether Mia1p NES mutation interfered with Alp14p binding. We investigated this question in the following ways. First, to check for direct protein-protein interaction we assayed whether recombinant MBP-tagged Mia1p or its NES mutant version expressed in bacteria could pull down Alp14p-myc from cellular extracts that were prepared from *mia1*Δ cells to avoid carrying over wild type Mia1p. MBP-Mia1p-MutNES4 could interact with Alp14p, albeit with a diminished efficiency as compared to the wild type Mia1p (Fig. 4C, 0.26∶1 ratio of Alp14p-myc pulled down by MBP-Mia1p-MutNES4 and MBP-Mia1p respectively). A similar result was obtained when the Alp14p-myc containing cellular extract was prepared from wild type cells (data not shown).

Secondly, to check whether Mia1p-MutNES4 mutant protein could, in principle, interact with Alp14p *in vivo*, we introduced a *pim1-1* temperature-sensitive mutation that abolishes function of the RanGEF, Pim1p, at 36°C [11] into *mia1-mutNES4*, *mia1*Δand wild type cells expressing Alp14p-GFP. *pim1-1* cells at the restrictive temperature fragment the NE during mitosis but are capable of chromosome segregation: the terminal arrest phenotype presents as septated cells with hyper-condensed chromosomes directly surrounded by cytoplasm. We reasoned that if Mia1p-MutNES4p and Alp14p were capable of interaction, release of Mia1p-MutNES4 from the nucleus would drive recruitment of Alp14p to microtubules. Indeed, we observed recruitment of Alp14p-GFP to microtubules in *pim1-1 mia1-mutNES4* (93% cells, n = 72) but not in *pim1-1 mia1*Δcells (0% cells, n = 72) shifted to 36°C (Fig. 4D), suggesting that while binding between Alp14p and Mia1p-MutNES4 was diminished (Fig. 4C) these proteins could interact when present in the same cellular compartment.

Third, we wondered whether overexpression of Mia1p-MutNES protein that partially retains Alp14p-binding activity could re-establish Alp14p nuclear accumulation during mitosis. For this purpose we expressed either mCherry-tagged Mia1p or Mia1p-MutNES4 under the control of *nmt81* promoter in *mia1-mutNES4 alp14-GFP* cells. As expected, ectopic overexpression of wild type Mia1p in this genetic background efficiently restored nuclear localization of Alp14p in mitotic cells (100%, n = 44). It also led to robust loading of Alp14p on interphase microtubules. Mia1p-mCherry now co-localized with Alp14p throughout the cell cycle (Fig. 4E). When overexpressed, Mia1p-MutNES4-mCherry accumulated in the nucleus at all stages of cell cycle, mimicking localization of this protein expressed under its native promoter (Fig. 4E). Importantly, overexpression of Mia1p-MutNES4-mCherry was able to drive Alp14p to the nucleus in mitosis, albeit with decreased efficiency as compared to the wild type (Fig. 4E, ∼20%, n = 44). The lower penetrance is consistent with decreased affinity between the two proteins. We concluded that extra copies of Mia1p-MutNES4 protein could partially alleviate the need for tight Alp14p binding to ensure nuclear localization of Alp14p during mitosis. Thus, mutating the Mia1p NES decreases interaction between Mia1p and Alp14p, indicating that the Alp14p binding site might overlap with the NES.

### Artificial accumulation of Alp14p in the nucleus partially rescues mitotic defects caused by mutation of the Mia1p NES

**Figure 5:**
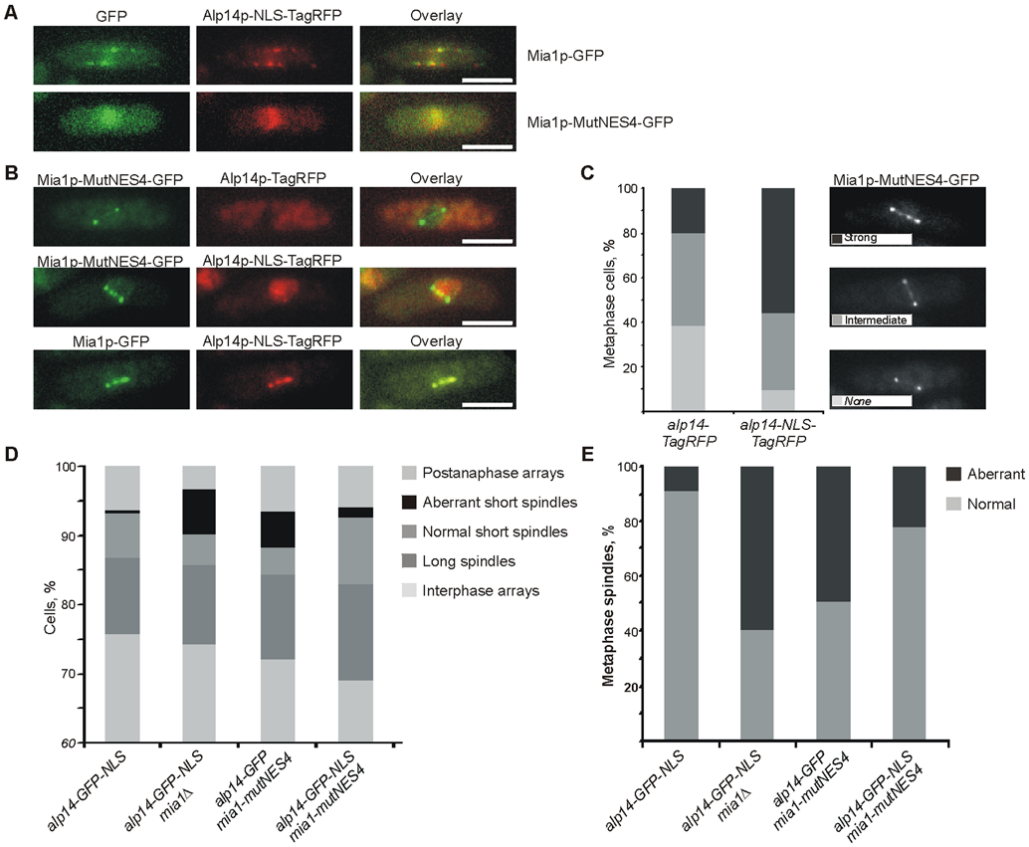
Artificial accumulation of Alp14p in the nucleus partially rescues the mitotic defects observed in Mia1p NES mutant cells. (A) Alp14p-NLS-TagRFP localizes to the nucleus during interphase, partially in *mia1-GFP*, and fully in *mia1-MutNES4-GFP* cells. Shown are single maximum intensity reconstructions of live cells. Scale bars = 5 µm. (B) Nuclear Alp14p-NLS-TagRFP partially restores localization of Mia1p-MutNES4-GFP to kinetochores during mitosis. Shown are single maximum intensity reconstructions of live cells. Scale bars = 5 µm. (C) Percentages of cells with various levels of Mia1p-MutNES4-GFP at kinetochores (n = 100 metaphase cells). Cells were binned into three classes based on intensity of GFP signal at kinetochores (strong, intermediate, none). Representative images are shown in the legend. (D) Percentages of aberrant mitoses and other cell cycle stages in asynchronous cell populations (n = 300 cells). Microtubules were immunostained with anti-α-tubulin antibody TAT-1. (E) Percentages of monopolar and broken short spindles (n = 100 spindles). Microtubules were immunostained with anti-α-tubulin antibody TAT-1.

To determine whether mitotic defects in *mia1-mutNES4* cells were indeed due to spatial separation between Alp14p and Mia1p, we attempted to drive Alp14p into the nucleus independently of Mia1p. To this end, we introduced the SV40 T-antigen derived NLS (PKKKRKV) in GFP- or TagRFP-tagged Alp14p and expressed these fusion proteins from the *alp14* chromosomal locus. Although Alp14p-NLS-TagRFP was still found on cytoplasmic microtubules in wild type cells, presence of the exogenous NLS was sufficient to partially accumulate the fusion protein in interphase nuclei (Fig. 5A, upper panel). This in turn appeared to elevate nuclear levels of Mia1p (Fig. 5A, upper panel, compare to control cells in Fig. 4B, upper panel). On the other hand, when the nucleocytoplasmic transport of Mia1p was disabled by NES4 mutation, both Alp14p-NLS-TagRFP and Mia1p-MutNES4-GFP strongly accumulated in the nuclei and were completely depleted from cytoplasmic microtubule arrays (Fig. 5A, lower panel). These data further support the hypothesis that it is the Mia1p NES that is required for the nucleocytoplasmic shuttling of Mia1p and Alp14p.

We then assessed whether nuclear Alp14p could restore Mia1p-MutNES4 localization to the kinetochores in mitosis. Mia1p-MutNES4-GFP was depleted from kinetochores in most cells expressing wild type Alp14p-TagRFP (Fig. 5B, upper panel, and 5C). Alp14p-NLS-TagRFP did not interfere with kinetochore localization of the wild type Mia1p, suggesting that the Alp14p-NLS fusion protein was functional (Fig. 5B, lower panel). Interestingly, presence of Alp14p in the nucleus was sufficient to partially restore recruitment of Mia1p-MutNES4-GFP to the kinetochores (Fig. 5B, middle panel and 5C). Consistently, Alp14p-NLS-TagRFP efficiently localized to the SPBs, along the spindle, and to the kinetochores in wild type cells but only weakly in *mia1-MutNES4* cells (Fig. 5B, middle panel). Partial rescue of Mia1p-MutNES4 localization during mitosis by Alp14p-NLS, together with weak spindle loading of Alp14p-NLS by Mia1p-MutNES4, could indicate that the Mia1p NES mutation does affect Mia1p-Alp14p binding. However, it appears that the residual interaction allows for partial restoration of Mia1p spindle localization.

Next we explored whether nuclear Alp14p could also rescue the spindle defects in *mia1-MutNES4* cells. Indeed, we observed a pronounced diminishing of aberrant mitoses in an *alp14-GFP-NLS mia1-MutNES4* genetic background as compared to *alp14-GFP mia1-MutNES4* cells (Fig. 5D and 5E). As expected, *alp14-GFP-NLS mia1Δ*cells exhibited strong spindle defects, confirming that the rescue by nuclear Alp14p depends on Mia1p function (Fig. 5D and 5E).

Collectively, our results suggest that the mitotic abnormalities caused by Mia1p NES mutation are, in part, due to defective nucleocytoplasmic shuttling of its partner protein, Alp14p. At steady state, lack of a functional NES in Mia1p sequence likely leads to spatial separation between Mia1p and Alp14p.

## Discussion

Microtubule regulators such as the TACC/TOG complex Mia1p/Alp14p [9], [10], EB1 protein Mal3p [12], CLASP protein Cls1p [13], [14], MAP65 protein Ase1p [15], [16] and others function in sustaining dynamics of both the mitotic spindle and cytoplasmic arrays. Therefore, cells have to localize microtubule regulators either to the nucleus or to the cytoplasm, depending on the cell cycle stage. While nuclear localization of large proteins and protein complexes requires specialized NLSs, at least two possibilities may account for the redistribution of MAPs once mitosis is complete. One could involve destruction of the nuclear pool of such protein and its *de novo* synthesis in the cytoplasm. This mechanism could be particularly important for timing spindle disassembly to mitotic exit, as in the case of the budding yeast Ase1p [17]. A delay in spindle disassembly could lead to the shortage of tubulin and MAPs available for cytoplasmic array assembly in organisms with complex cytoplasmic microtubule structures, such as *S. pombe*. Alternatively, the proteins could be actively exported, by Crm1p exportin or other specialized karyopherins. Here we report that a deficiency in Mia1p/TACC nuclear export results in its steady state nuclear accumulation. Consequently, depletion of Mia1p in the cytoplasm leads to manifestation of the *mia1*Δ-like interphase phenotype of abnormal microtubule bundles and often to bent cell shape. Our results underscore the importance of nuclear export of Mia1p/TACC and possibly other MAPs in remodeling microtubule arrays as cells return to growth.

Mia1p carries an NLS [6] and a strong “transferable” Crm1p-dependent NES motif L-X_3_-L/I/F/M-X_2_-L-X-L. During interphase, a combination of both signals results in a constitutive nucleocytoplasmic shuttling with the bulk of Mia1p located in the cytoplasm at steady state. The balance shifts dramatically during mitosis resulting in strong nuclear enrichment. What could be the mechanism for such regulation? The Mia1p NLS confers only a partial nuclear enrichment when fused to GFP and the nuclear-to-cytoplasmic ratio of GFP-NLS fluorescence intensity does not appear to increase during mitosis (data not shown). NLS activity *in vivo* might therefore depend on the sequence and possibly conformational context. Alternatively, NLS activity might not be subject to regulation and instead, cells could regulate the nuclear export of the protein. In this manner the relative strength of NLS and NES would determine the subcellular distribution of Mia1p. As NES-GFP does not equilibrate between the nucleus and cytoplasm during mitosis, indicating that Crm1p continues to function in nuclear export, we propose that it is the Mia1p NES activity that could be masked, intra- or intermolecularly, by cell cycle dependent post-translational modifications (PTMs) or, possibly, through interactions of the TACC/TOG complex with other proteins. Interestingly, mutating the Mia1p NES caused a reduction in Mia1p binding to Alp14p (Fig. 4 and 5). This could indicate that the NES partially overlaps with the Alp14p binding site. An intriguing possibility could be that under some circumstances (*e.g.* due to mitosis-specific PTMs) Crm1p and Alp14p compete for binding to Mia1p.

The subcellular distribution and possibly microtubule-organizing activity of other TACC proteins could be regulated in a similar manner. Human TACCs partially localize in interphase nuclei [18] suggesting that they could shuttle between the nucleus and cytoplasm. And while animal cells fragment the NE during mitosis it is the TACC activity that could be regulated by interaction with karyopherins [19]. In conclusion, we propose that exquisite regulation of the subcellular distribution of TACC/TOG complexes drives microtubule array remodeling as cells progress through the cell cycle.

## Materials and Methods

### *Schizosaccharomyces pombe* strains and constructs

*S. pombe* strains used in this study and their genotypes are listed in Table S1. We used standard genetic methods and S. *pombe* media for vegetative growth (EMM and YES). Genetic crosses and sporulation were performed on YPD agar plates. NES (LVIAMDQLNL) and MutNES (LVIAMDQANA) sequences were incorporated into the reverse (NES and MutNES) primers for generating the plasmids pREP81-GFP-NES and pREP81-GFP-MutNES. The expression of these GFP constructs was induced upon thiamine removal from the culture medium. Mia1-MutNES2 or Mia1-MutNES4 were generated using PCR-based targeted-mutagenesis, and were integrated into the original chromosome loci of *mia1* for expression under its endogenous promoter. Cold sensitive *crm1-809* strain was a kind gift from Dr. Simon Whitehall.

### Fluorescence microscopy and image analysis

Epifluorescence images were collected using a mercury lamp as illumination source with appropriate sets of filters on a Zeiss Axiovert 200 M microscope equipped with a CoolSnap camera (Photometrics) and Uniblitz shutter driver (Photonics, Rochester, NY, USA) under the control of Metamorph software package (Universal Imaging, Sunnyvale, CA, USA). Presented are the z-stack maximum projection images obtained using Metamorph build-in module.

Sum Z-projection images of z-stacks of 6 sections were used to determine nuclear and cytosolic fluorescence levels. First, the compartments were manually outlined and average fluorescence intensities were determined using in-built functions of ImageJ software (NIH, USA). Next, background signal obtained from non-fluorescent wild type cells for either compartment was subtracted. Statistical analysis was performed in Excel (Microsoft, USA).

Time-lapse fluorescence microscope images were generated on a Zeiss Axiovert 200 M microscope equipped with UltraView RS-3 confocal system: CSU21 confocal optical scanner, 12 bit digital cooled Hamamatsu Orca-ER camera (OPELCO, Sterling, VA, USA) and krypton-argon triple line laser illumination source under the control of Metamorph software package. Typically, we acquired a z-stack of 7 sections, through whole cells, spaced at 0.6 µm, every 30 or 15 seconds. Imaging was performed on *S. pombe* placed in sealed growth chambers containing 2% agarose YES media. Images were processed with Adobe Photoshop 7.0.

### Immunofluorescence techniques

Cells were fixed with cold methanol for 8 min and then spheroplasted using lysing enzymes in 1.2 M sorbitol in PBS. Spheroplasts were washed with PBS. PBAL buffer (1 mM sodium azide, 1% BSA, 100 mM Lysin-hydrochloride, 50 µg/ml Carbenicilin in PBS) was used for blocking and for incubation with anti-α-tubulin primary and secondary antibodies. Imaging was done on a Zeiss Axiovert 200 M microscope with appropriate sets of filters, and images were generated using a CoolSnap camera (Photometrics) and Metamorph software package (Universal Imaging, Sunnyvale, CA).

### Protein Expression and pull-down experiments

All reagents were obtained from Sigma unless otherwise stated. ORFs of full length Mia1 (1–474aa) and Mia1-MutNES4 were cloned into pAD2a vector (a kind gift from Andrej Didovik) that contains a maltose binding protein (MBP) gene upstream of the multiple cloning site. The plasmids were transformed into BL21 (DE3) competent cells carrying the pG-KJE8 chaperone plasmid. Overnight bacteria cultures were diluted in fresh medium, and the chaperone plasmid was induced with 0.5 mg/ml L-arabinose and 5 ng/ml tetracycline. Bacteria were grown at 37°C for 2 hours to reach log phase. Expressions of MBP or MBP-fusion proteins were induced by adding 0.5 mM IPTG and incubating at 24°C for another 3 hours. The bacteria were harvested by centrifugation, and the pellets were stored at −80°C or used immediately. The bacteria pellets were resuspended in ice-cold Buffer A (20 mM Tris-HCl pH 7.4, 400 mM NaCl, 1 mM EDTA, 10 mM β-mercaptoethanol) with freshly added protease inhibitors (1.3 mM Benzamidine, 1 mM PMSF, and protease inhibitor cocktail tablet, Roche Applied Science), and disrupted by sonication. Cellular debris was removed by centrifugation (16,000 x g, 10 min). The supernatant was applied to amylose resin (New England Biolabs) pre-equilibrated with buffer A and incubated for 1 h at 4°C. The amylose resin was washed three times in buffer A. For total yeast cell lysate, Alp14p-13myc expressing *mia1*Δ cells were grown to log phase, harvested and washed once with Buffer A. Cell pellet were homogenized with glass beads (425–600 µm) using Mini Bead Beater (Biospec) at 4°C. Cell lysates were harvested and centrifuged (16,000 x g, 10 mins) to remove cell debris and insoluble protein. Soluble proteins were incubated with washed amylose resins from above for 1 hour at 4°C before being washed 3 times with Buffer A and resuspended in SDS-loading buffer.

## Supporting Information

Figure S1 (A) Mia1p-GFP and Alp14p-TagRFP are retained in the nucleus of interphase crm1-809 cell at the restrictive temperature of 18oC. (B) NES derived from mia1 ORF (LVIAMDQLNL) drives exclusion of GFP from the nucleus. Pcp1p-mCherry is used as the SPB marker. Scale bars = 5 µm.
(0.84 MB TIF)

Table S1 Fission yeast strains used in this study
(0.07 MB DOC)

Movie S1 Interphase mia1-MutNES4 cells expressing Atb2p-GFP. Presented are the maximum intensity projections of 7 planes at 0.6 µm spacing, with time-lapse imaging performed at 30-second intervals.
(2.49 MB MOV)

Movie S2 Interphase wild type cells expressing Atb2p-GFP. Presented are the maximum intensity projections of 7 planes at 0.6 µm spacing, with time-lapse imaging performed at 30-second intervals.
(2.52 MB MOV)

Movie S3 Mitotic mia1-MutNES4 cells expressing Atb2p-GFP. Presented are the maximum intensity projections of 7 planes at 0.6 µm spacing, with time-lapse imaging performed at 30-second intervals.
(2.63 MB MOV)

Movie S4 Mitotic mia1-MutNES4 cells expressing Atb2p-GFP. Presented are the maximum intensity projections of 7 planes at 0.6 µm spacing, with time-lapse imaging performed at 30-second intervals.
(2.98 MB MOV)
