# Supplementary figures and images for: Nucleocytoplasmic Shuttling of the TACC Protein Mia1p/Alp7p Is Required for Remodeling of Microtubule Arrays during the Cell Cycle
Source: PLoS One. 2009 Jul 16;4(7):e6255. doi: 10.1371/journal.pone.0006255 (PMC2705800; doi:10.1371/journal.pone.0006255)

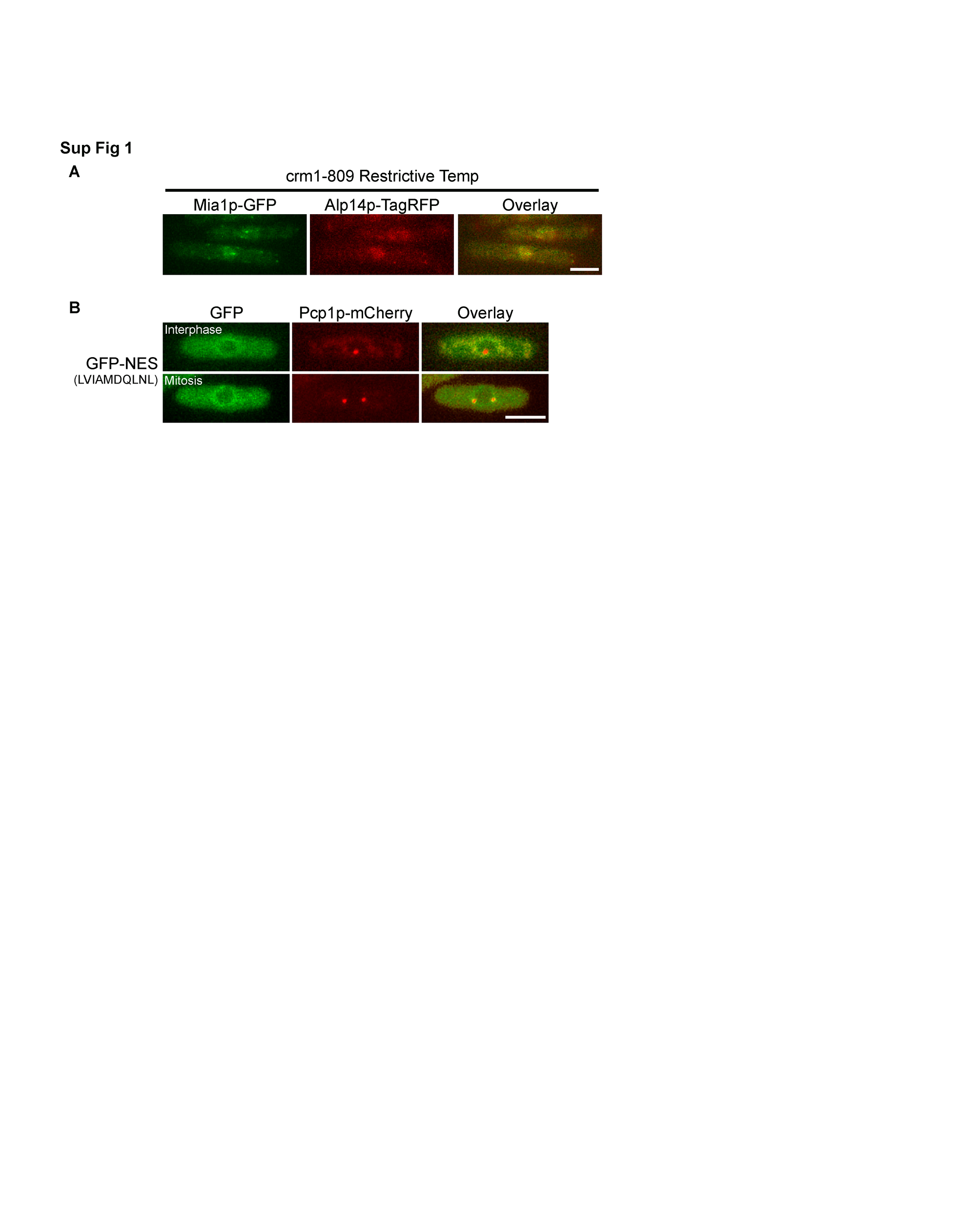

Supplement: Figure S1 — (A) Mia1p-GFP and Alp14p-TagRFP are retained in the nucleus of interphase crm1-809 cell at the restrictive temperature of 18oC. (B) NES derived from mia1 ORF (LVIAMDQLNL) drives exclusion of GFP from the nucleus. Pcp1p-mCherry is used as the SPB marker. Scale bars = 5 µm. (0.84 MB TIF) [file pone.0006255.s001.tif]
