# Supplementary material for: Nucleocytoplasmic Shuttling of the TACC Protein Mia1p/Alp7p Is Required for Remodeling of Microtubule Arrays during the Cell Cycle
Source: PLoS One. 2009 Jul 16;4(7):e6255. doi: 10.1371/journal.pone.0006255 (PMC2705800; doi:10.1371/journal.pone.0006255)
Supplement: Table S1 — Fission yeast strains used in this study (0.07 MB DOC) [file pone.0006255.s002.doc]

**Table S1**

| **No.** | **Genotype** | **Source** |
| --- | --- | --- |
| SO7 | Mia1p-13myc::ura4 *ade6-216 ura4D-18 leu1-32* | Lab collection |
| SO76 | pREP1-α-tubulin-EGFP *ade6-216 ura4D-18 leu1-32* | Lab collection |
| SO158 | Mad2p-GFP::ura4 *leu1-32* | Lab collection |
| SO1222 | Alp4p-GFP::ura4 ade6-210 ura4D-18 leu1-32 | Lab collection |
| SO1716 | Alp14p-GFP::ura4 *ade6-M216 ura4-D18 leu1-32* | Lab collection |
| SO2855 | Alp14p-13myc::ura4 *ade6-216 ura4D-18 leu1-32* | This Study |
| SO2919 | Pcp1p-mCherry::ura4 / Mia1p-GFP::ura4 *ade6-21X ura4D-18 leu1-32* | This Study |
| SO3651 | pREP81-EGFP / Uch2p-mCherry::ura4 *ade6-216 ura4D-18 leu1-32* | This Study |
| SO3653 | pREP81-EGFP-NES / Uch2p-mCherry::ura4 *ade6-216 ura4D-18 leu1-32* | This Study |
| SO3654 | pREP81-EGFP-MutNES / Uch2p-mCherry::ura4 *ade6-216 ura4D-18 leu1-32* | This Study |
| SO3657 | Mia1p-MutNES2-GFP::ura4 / Uch2p-mCherry::ura4 *ade6-21X ura4D-18 leu1-32* | This Study |
| SO3658 | Mia1p-MutNES4-GFP::ura4 / Uch2p-mCherry::ura4 *ade6-21X ura4D-18 leu1-32* | This Study |
| SO3660 | Mia1p-GFP::ura4 / Uch2p-mCherry::ura4 *ade6-21X ura4D-18 leu1-32* | This Study |
| SO3661 | Mia1p-MutNES2-GFP::ura4 / Pcp1p-mCherry::ura4 *ade6-21X ura4D-18 leu1-32* | This Study |
| SO3662 | Mia1p-MutNES4-GFP::ura4 / Pcp1p-mCherry::ura4 *ade6-21X ura4D-18 leu1-32* | This Study |
| SO3663 | Mia1p-MutNES4::ura4 / Pcp1p-mCherry::ura4 / Alp14p-GFP::ura4 *ade6-21X ura4D-18 leu1-32* | This Study |
| SO3669 | Pcp1p-mCherry::ura4 / Alp14p-GFP::ura4 *ade6-21X ura4D-18 leu1-32* | This Study |
| SO3702 | *pim1-1* / Mia1p-MutNES4::ura4 / Alp14p-GFP::ura4 *ade6-21X ura4D-18 leu1-32* | This Study |
| SO3705 | Mia1p-MutNES4::ura4 / Alp4p-GFP::ura4 *ade6-21X ura4D-18 leu1-32* | This Study |
| SO3708 | pREP1-α-tub-GFP / Mia1p-MutNES4::ura4 *ade6-216 ura4D-18 leu1-32* | This Study |
| SO3709 | pREP1-α-tub-GFP / *mia1*∆::ura4 *ade6-216 ura4D-18 leu1-32* | This Study |
| SO3833 | Alp14p-13myc::ura4 / *mia1*∆::ura4 *ade6-21X ura4D-18 leu1-32* | This Study |
| SO3865 | Alp14p-GFP::ura4 / Pcp1p-mCherry::ura4 / *mia1*∆::ura4 *ade6-21X ura4D-18 leu1-32* | This Study |
| SO3866 | Mia1p-GFP::ura4 / Uch2p-mCherry::ura4 / *crm1-809* | This Study |
| SO3869 | pREP1-α-tub-GFP / Pcp1p-GFP::kanR / Mia1p-MutNES4::ura4 *ade6-21X ura4D-18 leu1-32* | This Study |
| SO3889 | pREP1-α-tub-GFP / Pcp1p-GFP::kanR *ade6-21X ura4D-18 leu1-32* | This Study |
| SO3891 | *mia1*∆::ura4 / Mad2p-GFP::ura4 *ade6-21X ura4D-18 leu1-32* | This Study |
| SO3892 | Mia1p-MutNES4::ura4 / Mad2-GFP::ura4 *ade6-21X ura4D-18 leu1-32* | This Study |
| SO3893 | Mia1p-MutNES4::ura4 / *mad2*∆::ura4 *ade6-21X ura4D-18 leu1-32* | This Study |
| SO3975 | Alp14p-TagRFP::ura4 / Mia1p-GFP::ura4 *ade6-21X ura4D-18 leu1-32* | This Study |
| SO3976 | Alp14p-TagRFP::ura4 / Mia1p-MutNES4-GFP::ura4 *ade6-21X ura4D-18 leu1-32* | This Study |
| SO4068 | pREP81-Mia1p-mCherry / Mia1p-MutNES4::ura / Alp14p-GFP::ura4 *ade6-21X ura4D-18 leu1-32* | This Study |
| SO4083 | pREP81-mCherry / Alp14p-GFP::ura4 / Mia1p-MutNES4::ura4 *ade6-21X ura4D-18 leu1-32* | This Study |
| SO4084 | pREP81-Mia1p-MutNES4-mCherry / Alp14p-GFP::ura4 / Mia1p-MutNES4::ura4 *ade6-21X ura4D-18 leu1-32* | This Study |
| SO4511 | Alp14p-NLS-TagRFP::ura4+ Mia1-GFP::ura4+ *ade6-21X ura4D-18 leu1-32* | This Study |
| SO4512 | Alp14-NLS-TagRFP::ura4+ Mia1-MutNES4-GFP::ura4+ *ade6-21X ura4D-18 leu1-32* | This Study |
| SO4513 | Alp14-GFP-NLS::ura4+ *ade6-21X ura4D-18 leu1-32* h- | This Study |
| SO4514 | Alp14-GFP-NLS::ura4+ Mia1∆::ura4+ *ade6-21X ura4D-18 leu1-32* | This Study |
| SO4515 | Alp14-GFP-NLS::ura4+ Mia1-MutNES4::ura4+ *ade6-21X ura4D-18 leu1-32* | This Study |
